# Repetition and Aesthetic Judgment in Post-tonal Music for Large Ensemble and Orchestra

**DOI:** 10.3389/fpsyg.2021.673706

**Published:** 2021-06-07

**Authors:** Moe Touizrar, Anna Lena Knoll, Kai Siedenburg

**Affiliations:** ^1^Finnish Centre for Interdisciplinary Music Research, Department of Music, Art and Culture Studies, University of Jyväskylä, Jyväskylä, Finland; ^2^Department of Medical Physics and Acoustics, Carl von Ossietzky University of Oldenburg, Oldenburg, Germany

**Keywords:** repetition, post-tonal music, mnemonic affordance, musical coherence, psycho-aesthetics, aesthetic judgment

## Abstract

Post-tonal music often poses perceptual and cognitive challenges for listeners, potentially related to the use of relatively uncommon and unfamiliar musical material and compositional processes. As a basic compositional device, repetition affects memory for music and is structured by composers in very different ways across tonal and post-tonal musical repertoires. Of particular concern is whether post-tonal music exhibits mnemonic affordances that allow listeners to experience a sense of global coherence, and whether repetition correlates strongly with aesthetic judgment. Although previous research suggests that repetition impacts aesthetic preference, empirical research has not mapped out the relationship between repetition and aesthetic judgments across a broad set of post-tonal music. Presenting 14 excerpts, grouped into three categories: tonal, modernist, and post-1970, we observed that indications of repetitions in the music for a group of 60 listeners, with and without musical training, showed significant periods of interindividual synchronization. Aesthetic judgments were assessed by means of ratings for the following parameters: familiarity with the piece, confidence of repetition responses, judgments of affordances for easy listening, coherence, similarity of moments, and recognition, as well as listeners’ liking and interest. A principal component analysis (PCA) on the joint question data and the repetition responses suggested that two factors account for 92% of variance in the data. These factors were interpreted as dominated by aesthetic judgment and repetition strength. Linear mixed-effects regression indicated that repetition strength generally differed across excerpt category, with modernist excerpts featuring lower repetition strength compared to both post-1970 and tonal excerpts. Aesthetic preference, on the other hand, was lower for excerpts in both the modernist and post-1970 categories when compared to tonal excerpts. The analysis did not reveal difference in response behavior for repetition responses as a function of musical training, though it indicated higher preference of modernist excerpts with increasing levels of musical training. Overall, the results suggest that the two factors, aesthetic judgment and repetition strength, act as independent determinants in the experience of post-tonal music.

## Introduction

In the present study, we wish to contribute to a better understanding of the relationship between the perception of repetition, comprehensibility, and aesthetic judgment in the chronically understudied domain of post-tonal music. Post-tonal compositions often pose perceptual and cognitive challenges to listeners, potentially related to their novel and unfamiliar musical material and formal processes. Of particular concern for music perception and cognition is whether music of this type exhibits mnemonic affordances that allow listeners to experience a sense of similarity and coherence. As a basic compositional device, repetition affects memory for music and is employed by composers in very different ways across the post-tonal musical repertoire.

Listening encompasses a complex set of dynamic perceptual-cognitive processes that are geared toward both attending to the immediate features of an auditory event and to the relative integration of past events to form a continuous understanding (Levinson, 1997; London et al., 1999; Huovinen, 2013). Everyday acts of listening to music are adaptive and rooted in embodied cognitive mechanisms (Kozak, 2019; Reybrouck, 2020), and the processes that underlie the formation of intelligible musical experiences, for which repetition is often a key factor, must be approached from both music theoretical as well as cognitive perspectives. Music theorists identify the contoured patterning of the motive as the basic unit of coherence in the Western classical tradition (Carpenter and Neff, 1995; Van den Toorn, 1996; Boss, 2000). Motives are usually presented as compact memorable musical ideas at or near the beginning of a piece and are subsequently re-presented in various novel configurations that preserve the general identity of the original, while making important developmental changes to its structuring. In this way, motives serve as formal mnemonic signposts that are experienced across the temporal unfolding of musical pieces. The mind’s ability to establish and categorize connections between related motivic events engenders musical coherence and comprehension (Zbikowski, 1999).

### Repetition in Music Listening

Lamont and Dibben (2001) identify three cognitive factors that contribute to a listener’s understanding of motivic coherence: musical experience, familiarity – both with general styles of music and within specific pieces – and the degree of complexity in the surface features of a given auditory event. Repeated units of music accrue in experience and can produce habituation – a decline in responsiveness as the novelty of a stimulus recedes relative to exposure. The attentive focus of the mind relaxes as sonic patterns take on increasing familiarity. In a review of previous findings, Huron (2013) lists five factors that influence the speed with which habituation occurs. Firstly, the number of presentations of a given stimulus has a direct impact on the degree to which the mind assesses the importance of an event. Secondly, the rate of repetition helps to determine both the degree of imprint and the relative importance of the event; too few presentations spaced at too wide an interval will lessen the cognitive importance of a stimulus and the subsequent anticipation of its re-presentation. Thirdly, the relative predictability of a stimulus can affect the speed at which habituation takes effect. Fourthly, the relative prominence and energetic magnitude of the stimulus has a direct bearing on its integration and on how quickly its repetition leads to habituation. Energetic stimuli often resist habituation for a longer period than lower energy stimuli. Finally, the particular biographical history of the listener – whether they have a high degree of experience with a given type of musical stimulus or style – plays a determining role in their ability to both track and predict repetitions. Repeated exposure increases habituation.

During listening, similarity judgments depend primarily on the relative saliency of shared features perceived between auditory cues and on the listener’s ability to organize representations, both present and past, into meaningful categories (Cambouropoulos, 2001). Within complex auditory scenes, similarity judgments for polyphonic textures are based on perceived differences in features such as amplitude, articulation, textural density, and gestural contour (Lamont and Dibben, 2001). Building upon early work on prototype theory by Rosch (1975) and Tversky (1977), and subsequent alternatives proposed by Murphy and Medin (1985), two primary modes of categorization have been proposed: perceptual equivalence (prototypical categorization) and theory-based classification (Cambouropoulos, 2001; Deliège, 2001a,b, 2007; Lamont and Dibben, 2001). Prototypical approaches to musical repetition hold that the presentation and initial few repetitions of a motive represent the privileged exemplar of a category, to which all subsequent similar repetitions, however, much altered, are understood to belong. In the context of music cognition, theory-based categorization involves prior knowledge of a given style of music acquired through acculturation. According to this view, categorization involves not only shared surface-level attributes between items (as in prototype models), but also an underlying conceptual knowledge that helps to select which items in a given scene should be attended to (Lamont and Dibben, 2001).

Echoing the theory-based view of categorization, Deliège (2001b) notes that listeners who are already familiar with a given piece of music employ functional categorization, where pre-existing concepts formed by previous exposure allow contextual knowledge to supersede surface-level similarity. Alternatively, in cases of initial exposure to unfamiliar music, Deliège (2001b) proposes that categorization occurs in real time as a prototypical comparison between present and past events, where similarity in features determines categorical grouping. Furthermore, the pairwise comparison of features relative to an initial prototype ascribes a highly mnemonic mode of cognition for music listening, at least where the repetition of material functions as the primary mode of musical organization. In these contexts, repetition engenders saliency by focusing attention on both what is at hand within an auditory scene as well as on the relationship of the present auditory event with those that have recently past. It is noteworthy here that Taher et al. (2016) demonstrate compelling evidence suggesting that when presented with tonal two-part contrapuntal textures, listeners are susceptible to a rapid type of habituation capable of guiding attention *away from* repeated motives in one voice and toward novel information in another after just a single repetition.

### Aesthetic Judgment

Two primary factors have been shown to influence aesthetic judgments: familiarity through repeated exposure and the level of complexity displayed by a given stimulus (Hargreaves, 1984; North and Hargreaves, 1995). The “mere exposure” effect describes the positive increase in aesthetic judgment of music based on prior exposure, memory formation, and the accumulation of perceptual representation structures (Peretz et al., 1998). Repeated exposure to motives or other forms of recursion within musical works has been positively correlated to liking. Tillman and Bigand (1996) segmented two tonal piano pieces by Bach and Mozart, together with a post-tonal piece by Schoenberg into short chunks lasting approximately 6 s. Segments were linked into longer strands that either preserved the composer-intended order, or that reversed that order, but included all between-segment repetitions. The reversed order condition preserved the internal structure and ordering of each chunk, while obliterating the formally chained structure of the chunks as a larger ordered sequence. Non-musician participants rated the strands (either original or inverted) for musical expressiveness using 29 semantic scales, including ratings for coherence. Ratings for expressiveness and coherence across conditions showed no significant differences for the tonal music, and only minimal difference for the post-tonal music. Tan et al. (2006) had participants listen to 1-min excerpts of tonal piano music using two stimuli conditions: (1) intact excerpt and (2) patchworked hybrids of three 20-s excerpts from music by different composer linked together seamlessly without regard for similarity in thematic material, or structural parameters such as harmony, key, and tempo. Repeated hearings led to linear increase to cohesion and liking ratings for the patchwork compositions, while repeated exposure to intact stimuli led to decreased ratings.

### Mnemonic Affordance and Aesthetic Intention in Post-tonal Music

The various perceptual and cognitive processes and schemata in operation during listening to tonal music have long been studied (Meyer, 1956; Lerdahl and Jackendoff, 1983; Krumhansl, 1990). Whereas tonal music is organized using structural features derived from the major-minor scale system that are shared across pieces and across historical style periods, post-tonal music lacks a similar degree of shared structuring and inter-stylistic uniformity. Early post-tonal music was freely structured from piece to piece and composer to composer (c. 1909–1919). Breaking from tonal conventions, where repetition and intelligibility are key factors in the perceived articulation of musical form, composers of the Second Viennese School, such as Arnold Schoenberg, Alban Berg, and Anton Webern (to name only the most prominent), re-imagined the very organizing principles of music. Their search for a shared structure that could replace tonal organization led to a subsequent period defined by the strict serial ordering of pitch classes, commonly referred to as “serialism” (c. 1919–1937).

Following the upheaval of the Second World War, a new approach to the post-tonal structuring of musical materials emerged, termed “total serialism” (c. 1945) for its serialization of additional musical parameters such as rhythm and dynamics. The tendency toward procedural evisceration of easily perceived repetition in order to create new forms of music was a key aesthetic goal of post-war modernist composers, many of whom pointed to a previous general precedent in the music of the Second Viennese School, and to the early post-tonal music of Anton Webern in particular (Erwin, 2021). However, several other key characteristics of post-war modernist music serve to shed light on the general renouncement of conspicuous repetition by composers of this period. Whether dogmatically serial in construction or not, modernist composers often eschew various cognitively salient facets of music that engender easy listening and a familiar sense of continuity and flow, including the use of octaves, consonance, and simple rhythmic grouping (Koivisto, 1996; Barton, 2012). Rather, modernist music is largely anti-thematic (and thus anti-repetitive) and places emphasis on the formal organization of various parameters of sound itself, such as pitch, duration, dynamics, and timbre.

For the post-war modernists, composition was seen as an experimental but highly structured process, one that underscored the acts of *making* and *analyzing* a work of music (Griffiths, 2011). That is to say, unity was privileged at the level of construction and made evident by the analysis of a work, but the music itself was composed intentionally to resist any easy-to-follow presentation of its organizing principles at the level of experience. Perhaps unsurprisingly, coherence as understood in tonal forms of music therefore becomes an easy and early casualty of the modernist artistic agenda to occult organization, wrenching musical variation away from a centralized and salient prototype such as a motive or theme. Difference was privileged over repetition (Campbell, 2013), resulting in the divorce of any strong sense of a work’s internal unity from the act of listening. One conspicuous difference between music composed during the modernist period and, generally speaking, music composed post-1970, is the latter period’s aesthetic reaction to and subversion of post-war modernism in the form of increased emphasis on experiential coherence (Hutchinson, 2016), often without forsaking structural rigor (Losada, 2009). Rather, the post-1970 era of post-tonal music articulates an affinity for music that affords novel forms of mnemonic salience (Pasler, 1993).

### Empirical Studies on Post-tonal Music

The majority of empirical studies of similarity in post-tonal music have been conducted using music written for a single instrument. Krumhansl (1991) studied the function of memory for so-called “surface features” in the absence of the normative inter-opus cognitive schemata used for tonal music. She focused on the abstraction of features in post-tonal music and the ability to identify their characteristics, and to generalize surface features in subsequent passages. She found that listeners encoded and remembered a large amount of surface details in listening to a total serial piece for solo piano by Olivier Messiaen. Moreover, her results demonstrate that listeners abstract and retain knowledge regarding surface features and can accurately identify unfamiliar sections of the music as belonging to the same piece. Using eight 1-min long excerpts of pieces by Luciano Berio and Elliott Carter (each scored for a single instrument), Margulis (2013) had participants rate both original and modified recordings of post-tonal music. Modification involved artificially inserting repetitions into the original stimuli without specific regard for artistic or aesthetic considerations. Participants without previous experience of post-tonal music found the music to be more enjoyable and interesting when the excerpts contained added repetitions, regardless of whether the inserted repetition was immediate or placed later in the timeline of the excerpt. Importantly, as Margulis (2013, p. 54) notes,

*End-of-session debriefings revealed that listeners were unaware that they had been exposed to the same excerpts in different conditions – they neither recognized that they had reheard particular examples in several forms, nor that the degree of internal repetition was varying from excerpt to excerpt. Thus, the differences in enjoyment ratings did not stem from conscious awareness of the relevant manipulations. Rather, higher degrees of repetition were associated with higher enjoyment ratings in such a way that listeners were unaware of this association*.

Although few studies to date examine post-tonal music composed for ensemble or orchestra, those that do have found evidence for the influence of timbre and changes to instrumentation on the identity of musical materials and the perception of similarity, the classification of related motives, and the experience of either repetition or segmentation in the large-scale unfolding of musical form (McAdams et al., 2004; Poulin-Charronnat et al., 2004; Taher et al., 2018).

### The Present Study

Given the limited number of studies on post-tonal music and the direct relationship between repetition, musical form, and aesthetic preference, important questions remain unarticulated. How do the various strategies for repetition across styles of post-tonal music affect listening? How are non-verbatim repetition and aesthetic judgment related for a diverse population of listeners? And do previous findings with regard to aesthetic judgment hold up in ecologically valid paradigms? In the present experiment, 60 participants with diverse musical backgrounds were presented the first 3 min of all pieces in random order and were instructed to respond by pressing a button whenever they encounter a repetition in the music, where repetition is defined as something sounding familiar or self-similar within the piece. A second task asks participants to rate each excerpt for effects such as memorability, perceived coherence, and self-similarity. We predicted that (a) the total number of responses as well as the inter-participant agreement of responses would be high for classes I (tonal) and III (post-1970) and low for class II (modernist), and (b) that the total number of responses as well as the inter-participant agreement of responses would correlate with ratings for both overall memorability and coherence of the individual excerpts as well as ratings of aesthetic preference for these excerpts.

## Materials and Methods

### Participants

Sixty listeners participated in the experiment, who were recruited as part of two groups: one group with and another group without experience in playing a musical instrument. The former group consisted of 30 participants (10 males, 20 females) who reported having played at least one musical instrument for more than 2 years. Participants in this group had a mean age of *M* = 24.6 years (STD = 3.3, range: 19–32) and had played their primary musical instruments for *M* = 9.2 years (STD = 6.3) and had received *M* = 4.5 years of music theoretical instruction (STD = 3.0). We measured musical training using the corresponding self-report inventory of the Goldsmiths Musical Sophistication Index (MSI, Müllensiefen et al., 2014), see the Appendix for details. The MSI yielded mean scores of 31.4 (STD = 20.3, range: 6–80) for the musician participants. One participant reported mild hearing loss, all other participants reported normal hearing. Another 30 participants (eight males, 22 females) reported not to have played a musical instrument for more than 2 years and had a mean age of M = 24.2 years (STD = 3.0, range: 19–33). In this group of participants, one participant reported moderate hearing loss. All participants received monetary compensation.

### Stimuli

Fifteen musical excerpts were selected as stimuli for the present experiment. Excerpts were obtained from www.youtube.com. All stereo clips were peak-normalized in amplitude and converted to mp3 format (320 kbit/s). Of the 15 excerpts, three excerpts were from the tonal repertoire. Due to a technical problem, however, major portions of the data from one of the tonal excerpts (Manuel de Falla: *Ritual Fire Dance*) were lost. For that reason, only the data from the 14 other excerpts will be considered. Table 1 provides information about the respective composers, titles, year, the performers and the duration and temporal placement of the excerpt used in the experiment.

**Table 1:** Excerpt information.

Composer	Title	Year	Performer	URL	Duration	Timing
**I – Tonal**						
Tchaikovsky	Violin Concerto (movement III)	1878	Janine Jensen, Deutsche Radio Philharmonie, Christoph Poppen	https://www.youtube.com/watch?v=KrVMmRWzRSM	3:06	0:00–3:06
Grieg	Holberg Suite (movement I)	1884	‘A Far Cry’ String Ensemble	https://www.youtube.com/watch?v=dFEBTbNs4yk	2:40	0:00–2:40
**II – Modernist**						
Stockhausen	Gruppen	1957	Berliner Philharmoniker, Friedrich Goldman (I) Claudio Abbado (II) Marcus Creed (III)	https://www.youtube.com/watch?v=CZ7jpKh_UF0	3:06	0:00–3:06
Xenakis	Achorripsis	1957	Luxembourg Philharmonic Orchestra, Arturo Tamayo	https://www.youtube.com/watch?v=rEyqJPW3Hi8	2:55	0:00–2:55
Ligeti	Apparitions	1959	Berlin Philharmonic Orchestra, Jonathan Nott	https://www.youtube.com/watch?v=pCS8DJJnxOE	3:07	0:00–3:07
Stravinsky	The Flood, (movement III)	1962	London Sinfonietta, Oliver Knussen	https://www.youtube.com/watch?v=ISCnHvosib4&list=OLAK5uy_klRrg8cPiONrF_ekQtLOIf5P0Xv2pC3Zg&index=6	2:34	0:00–2:34
Penderecki	Fluorescences	1962	Polish National Radio Symphony Orchestra, Antoni Wit	https://www.youtube.com/watch?v=DBbSZD2IkJI	3:01	0:00–3:01
Boulez	Figures-Doubles-Prismes	1968	BBC Symphony Orchestra, Pierre Boulez	https://www.youtube.com/watch?v=SKEBBKQ82_8&t=0	3:00	0:00–3:00
**III – Post-1970**						
Grisey	Partiels	1975	Asko Ensemble, Stefan Asbury	https://www.youtube.com/watch?v=1v7onrjN6RE&list=RD1v7onrjN6RE&start_radio=1&t=0	3:06	0:00–3:06
Dutilleux	L’arbre des songes	1985	Olivier Charlier, BBC Philharmonic, Yan Pascal Tortelier	https://www.youtube.com/watch?v=EDVFNh7MDQk	3:14	0:00–3:14
Pärt	Fratres	1991	Antal Eisrich and Miklós Kovács, Strings of Hungarian State Opera Orchestra, Tamás Benedek	https://www.youtube.com/watch?v=UIeIRghsD_k	3:07	0:00–3:07
Hurel	Six miniatures en trompe-l’œil, (movement III)	1991	Ensemble Intercontemporain, Pierre Boulez	https://www.youtube.com/watch?v=IT4jQilFq8o	2:50	7:12–10:02
Boulez	Sur Incises	1998	Ensemble Intercontemporain, Matthias Pintscher	https://www.youtube.com/watch?v=HCQI6Wu3QxE	3:08	0:00–3:08
Romitelli	Flowing down too slow	2001	Musiques Nouvelles, Jean-Paul Dessy	https://www.youtube.com/watch?v=Xg5UQVa5CBA	3:02	0:00–3:02

The chosen excerpts were taken from ensemble and orchestra pieces that fall into one of three categories: *tonal*, *modernist*, and *post-1970*. Tonal pieces (by Pyotr Ilyich Tchaikovsky and Edvard Grieg) were selected for their clarity of repetition and comprehensibility. Modernist pieces (by Karlheinz Stockhausen, Iannis Xenakis, György Ligeti, Igor Stravinsky, Krzysztof Penderecki, and Pierre Boulez) were selected as representative pieces of the post-war era of musical modernism (the late 1940’s, the 1950’s, and the 1960’s). These pieces are generally characterized as difficult to follow by non-specialist audiences. Modernist music generally lacks clear repetitive structuring and do not often utilize a tonally-centered or consonant musical language. While the selected pieces for the post-1970 category (by Gérard Grisey, Henri Dutilleux, Arvo Pärt, Philippe Hurel, Pierre Boulez, and Fausto Romitelli) do not themselves constitute a particular style-grouping, they do share two general structural affinities relative to the modernist pieces: a more pronounced use of repetition and a noticeably more consonant musical language. The authors acknowledge that the selection of pieces is not, and indeed could not be representative of the many inter- and intra-stylistic idiosyncrasies, artistic agendas, and other genre-oriented determinants of music within and between the chosen categories.

### Procedure

The experiment was implemented using the test platform www.testable.org. Participants were recruited from the online job board of the University of Oldenburg and received a private link that provided access to the experiment. They were instructed to listen with headphones to the presented experimental stimuli. For every stimulus, participants were first asked to indicate repetitions in the music as the music unfolded and second to respond to a set of eight questions. Specifically, they received the following instruction: “Please press the spacebar whenever you have the impression that some aspect of the music is repeating. This repetition does not need to be exact. Rather, you should press the spacebar whenever you feel a sense of repetition in the music. You should indicate a repetition at least once per excerpt.” After the music had ended, participants received the following set of questions that were to be answered on scales from one to five: (Q1) Have you heard the piece before? (certainly not – certainly yes); (Q2) How confident are you in your ability to identify repetitions in this excerpt? (highly confident – highly unconfident); (Q3) How easy was it to follow this music? (very difficult – very easy); (Q4) How coherent was the piece? (highly coherent – highly incoherent); (Q5) How similar were individual moments of the piece? (highly similar – highly dissimilar); (Q6) Do you think you will recognize this piece if you hear it a week from now? (certainly yes – certainly no); (Q7) Did you like the piece? (certainly yes – certainly no); (Q8) Did you find the piece interesting? (certainly yes – certainly no). At the end of the experiment, there was a demographic questionnaire. The research reported in this manuscript was carried out according to the principles expressed in the Declaration of Helsinki and was approved by the ethics board of the University of Oldenburg.

### Data Analysis

The relative timings of the spacebar taps were represented at a sampling rate of 1,000 Hz. Each tap was converted into a rectangular function of height one and with a width of 1 s, centered at the timepoint of the original tap. That is, per participant and per piece, repetition responses consisted of a sequence of zeros and ones that encoded indicated repetitions with a temporal granularity of 1 s. In order to obtain a measure of inter-participant synchronization, these series were averaged across participants. The resulting time-series of inter-participant synchronization corresponds to the proportion of participants that simultaneously indicated a repetition at a given point in time with a tolerance of plus/minus 1 s. Note that these data capture more fine-grained patterns of synchronization compared to histograms with, say, 1-s bins, because the latter approach does not appropriately represent closely spaced patterns at time points with non-integer periodicity. Significant periods of synchronization were assessed *via* bootstrapping: for every stimulus, synchronization time-series were computed for 60 randomly selected participants (drawn 1,000 times with replacement). If for a given time point, the first percentile of the bootstrapped distribution exceeded listeners’ mean response rate across the entire excerpt, the time point was considered to exhibit significant inter-participant synchronization.

Two summary measures were computed from the repetition data: First, the raw number of indicated repetitions per participant and piece was computed (“#Rep”). Next, the duration of segments with significantly synchronized responses relative to the overall stimulus duration was considered (“Prop. Sync.”). The data from questions Q1–Q8 were analyzed descriptively and by providing 95% CIs for means across participants. In order to explore the major factors underlying participants’ responses, the data from variable (i.e., questions) Q1–Q8 together with the variable #Rep were z-normalized by participant and by variable, before a principal component analysis (PCA) with factor rotation was computed. The Prop. Sync. variable was not used in the PCA, because it yielded data on a group level and was not specific to individual participants. Finally, a linear mixed model (LME) was run to confirm effects of stimulus category (tonal, modernist, post-1970) and musical training on the two major factors identified by the PCA. The data analysis was implemented in MATLAB.^1^ The LME was implemented in R using the lme4 package (Bates et al., 2014).

## Results

**Figure 1:**
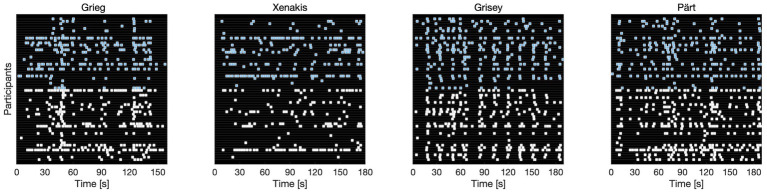
Repetition response data from all 60 individual participants for four selected stimuli. White and blue dots correspond to participants with and without musical training, respectively.

Figure 1 shows the repetition responses (as indicated by individual depressions of the space bar) for all 60 participants for four selected examples (blue and white dots correspond to participants with and without music training, respectively). As indicated by the figure, individual excerpts are characterized by different response rates; for instance, responses to Xenakis’ *Achorripsis* are sparser compared to the other three examples. Whereas Xenakis does not visually exhibit points where responses appear to be synchronized across participants, Grieg’s *Holberg Suite* and Pärt’s *Fratres* show multiple timepoints where this appears to be the case. Even more clearly, Grisey’s *Partiels* exhibits a quasi-periodic structure of strong synchronization across almost all participants. This is not to say that there is no individual variability. In fact, there are substantial differences in response rates across participants: 29 participants had average response rates of less than five responses per piece, whereas the other half of participants had mean rates of 12.2 responses per piece (max = 35).

However, as is already visible in the displayed individual data in Figure 1, for the tapping data there were no indicative differences between the two groups of participants with and without musical training. Time series of synchronous responses were highly similar for both groups such that the distribution of differences between groups significantly deviated from zero only for less than 1 % of time points (*M* = 0.5%, STD = 0.4%, max = 1,1%).

**Figure 2:**
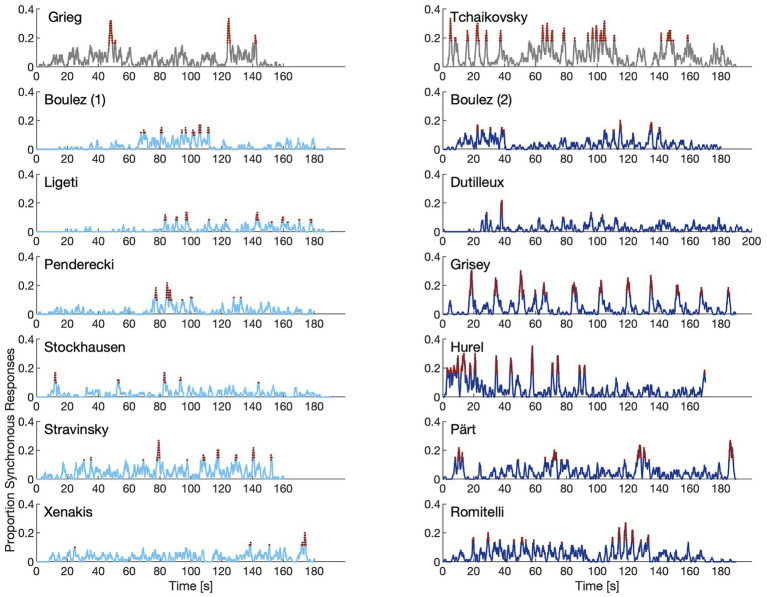
Proportion synchronous responses across participants (with a tolerance of plus/minus 1 s). Red portions of the graphs indicate time points where the synchronization significantly differed from chance.

Figure 2 displays the proportion of synchronous responses across participants and the time points (in red), where significant synchronization across participants is indicated (at an alpha level of 0.01). These response signals show that for all excerpts in all categories, there are time-points of significant inter-participant synchronization. The excerpts of the modernist stimulus set tend to exhibit a sparser distribution of synchronization periods compared to post-1970 excerpts. Furthermore, individual excerpts show distinct response profiles, which becomes particularly pronounced when comparing response profiles within categories of excerpts, such as between Grieg and Tchaikovsky or Grisey and Hurel.

**Figure 3:**
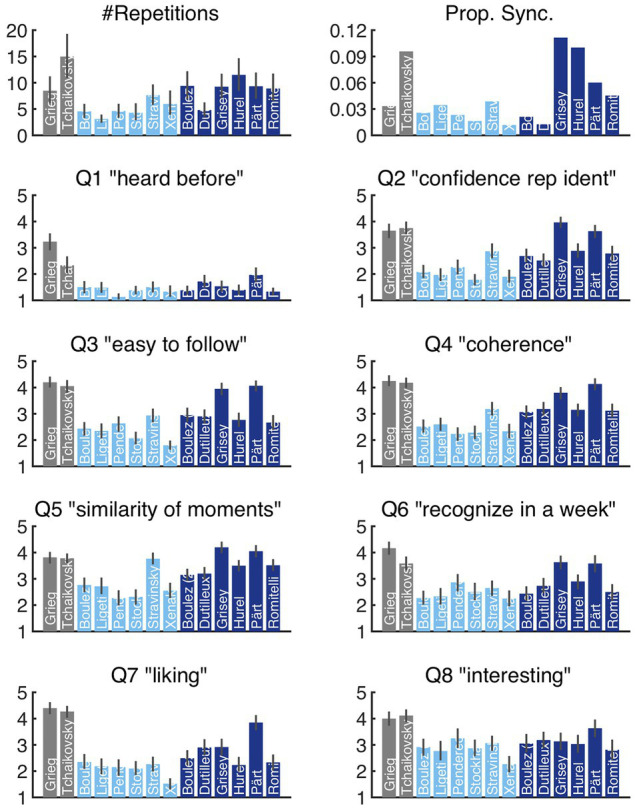
Overview of responses in terms of the number of repetitions (#Repetitions), the proportion of time that a stimulus generated significant synchronization (Prop. Sync.), and responses to the eight questions Q1–Q8. Error bars correspond to 95% CIs.

Two indices of the repetition data (average #repetition responses, proportion of significant synchronization) plus the responses to questions Q1–Q8 are given in Figure 3. Indices of repetition responses show substantial differences across stimuli. Whereas there is a medial number of repetition responses and rather low proportion of synchronized responses for Grieg, Tchaikovsky yields many more as well as more strongly synchronized responses. The proportion of synchronization is relatively low for modernist excerpts but varies strongly for post-1970 excerpts with the highest values for Grisey, followed by Hurel, Pärt, and Romitelli. Considering the questions Q1–Q8, all but the two tonal excerpts were rated as unfamiliar in Q1. Within the modernist category of excerpts, Stravinsky stands out by receiving particularly high scores in Q2 (confidence rep. ident.), Q4 (coherence), and Q5 (similarity of moments). From the category of post-1970 excerpts, Grisey and Pärt receive particularly high scores in questions Q2–Q6. When it comes to Q7 (liking), however, only Pärt receives scores that approach those of the tonal excerpts by Grieg and Tchaikovsky.

**Figure 4:**
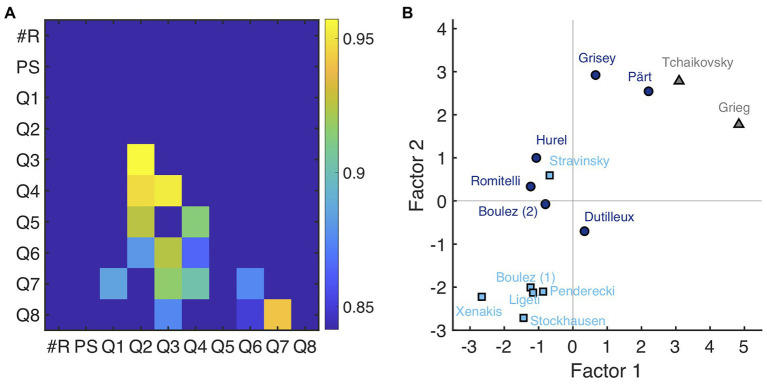
**(A)** Linear correlation matrix displaying significant correlations according to Bonferroni-Holm correction for multiple comparisons. **(B)** Coordinates of individual excerpts according to Factor 1 (aesthetic preference) and Factor 2 (repetition strength) derived by principal component analysis (PCA) and varimax rotation.

To explore the underlying structure of these 10 variables (see Figure 4A, for a correlation matrix), a PCA on the data averaged across participants was computed. The first two components accounted for 92% of the variance in the data and exhibited a clear knee point in the scree plot, which is the reason why a two-dimensional representation was adopted. To increase interpretability, components were rotated using the varimax rotation. The resulting rotated factors are displayed in Table 2. Results indicate strong loadings of questions Q1 (familiarity), Q7 (liking), and Q8 (interest) on Factor 1, highlighting that this first factor is dominated by aspects of familiarity and aesthetic preference. The number of repetitions together with Q5 (similarity of individual moments) strongly load on Factor 2, suggesting that this second factor reflects the perceived strength and frequency of repetitions in the music. Note that Factor 2 (repetition strength) robustly correlated with the proportion of synchronous responses, *r* = 0.83, CI: [0.54, 0.96], *p* < 0.001 [whereas there was only a marginal correlation for Factor 1, *r* = 0.51, CI: (−0.03, 0.81), *p* = 0.065]. This confirms that excerpts with higher scores of repetition strength also featured more moments of inter-subjective agreement about the presence or absence of repetitions. Taken together, the analysis indicates that the present data are determined by the two major factors of aesthetic preference and perceived repetition strength.

**Table 2:** First two components of the PCA after varimax rotation.

Variable	Factor 1	Factor 2
#Rep	−0.20	0.59
Q1	0.54	−0.13
Q2	0.09	0.42
Q3	0.26	0.26
Q4	0.21	0.31
Q5	0.06	0.52
Q6	0.34	0.15
Q7	0.45	−0.03
Q8	0.47	−0.02

The top three highest loadings per factor are marked in bold font. “#Rep” corresponds to the average number of repetitions per excerpt.

Figure 4B provides the coordinates of the mean responses for each piece with respect to Factors 1 and 2. Modernist excerpts tended to cluster according to both low aesthetic preference and repetition strength. An exception is the excerpt by Stravinsky, which scored higher in terms of repetition strength. Post-1970 excerpts had higher scores with respect to repetition strength (Factor 2). Most notably, Grisey and Pärt had similar scores in terms of repetition strength compared to the tonal excerpts. In terms of aesthetic preference, however, Tchaikovsky and Grieg yielded higher scores. Overall, three clusters emerged from the PCA: modernist excerpts with low aesthetic scores and low repetition strength, post-1970 excerpts with low aesthetic preference scores but medial repetition strength scores, and tonal or post-1970 excerpts with rather high aesthetic preference scores and high repetition strength.

In a final step, the observation regarding the effects of stimulus category on aesthetic preference and perceived repetition strength was sought to be confirmed by means of regression modeling. Two separate LME were set up to test the fixed effects of stimulus category (I: tonal, II: modernist, III: post-1970) and MSI scores on aesthetic preference (Factor 1) and repetition strength (Factor 2). The data for these two dependent variables was derived by projecting the data of individual participants on the rotated factors that were derived from the PCA (see the Supplementary Material for a visualization). The random effects structure of the LME consisted of by-participant intercepts and slopes for the stimulus category and by-item (i.e., stimulus) intercepts.

**Figure 5:**
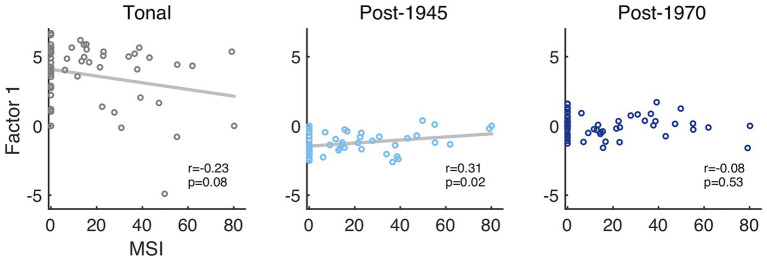
Scatter plot of the Gold-Musical Sophistication Index (MSI) of musical training (Müllensiefen et al., 2014) and Factor 1 (aesthetic preference).

Considering the aesthetic preference factor, estimated marginal means (95% CIs in square brackets) of the three categories were 1.38, CI: [0.79, 1.97], for tonal excerpts, −0.47, CI: [−0.80, −0.13], for the modernist excerpts, and 0.01, CI: [−0.33, 0.34], for the post-tonal excerpts. With aesthetic preference as dependent variable in the LME, there were significant differences between modernist and tonal excerpts [*β* = −1.85, CI: (−2.45, −1.24), *p* < 0.001] and between post-1970 and tonal excerpts [*β* = −1.38, CI: (−2.00, −0.76), *p* = 0.001], but only a marginal effect of musical training as measured by the MSI [*β* = −0.16, CI: (−0.34, 0.02), *p* = 0.091]. There was an interaction of the factor of musical training and the factor contrasting tonal and modernist excerpts [*β* = 0.24, CI: (0.03, 0.44), *p* = 0.028], see the Supplementary Material for the full set of statistics. As indicated in Figure 5, this interaction effect is also visible in a trend toward a negative correlation between participants’ MSI scores and their raw scores along Factor 1 (averaged across excerpts) for tonal excerpts, *r* = −0.23, *p* = 0.08, together with a weak positive correlation for modernist excerpts, *r* = 0.31, *p* = 0.02. That is, in general the stimulus categories of modernist and post-1970 excerpts were less preferred compared to the tonal excerpts, but participants with more formal musical training appeared to show slightly higher aesthetic preference for modernist excerpts compared to participants with less musical training.

Considering the repetition strength factor, estimated marginal means were 0.82, CI: [0.10, 1.54], for the tonal excerpts, −0.64, CI: [−1.05, −0.22], for the modernist excerpts, and 0.36, CI: [−0.05, 0.78], for the post-1970 excerpts, indicating least repetition strength for the modernist excerpts. With repetition strength as dependent variable in the LME, there was a significant effect of modernist excerpts compared to tonal excerpts [*β* = −1.45, CI: (−2.20, −0.71), *p* = 0.003], but repetition strength did not differ for post-1970 excerpts compared to tonal excerpts [*β* = −0.46, CI: (−1.20, 0.28), *p* = 0.25] and there also was no effect of musical training [*β* = 0.02, CI: (−0.17, 0.12), *p* = 0.74] and no interaction effect, see the Supplementary Material for the full set of statistics. Hence, modernist excerpts showed significantly less repetition strength compared to tonal and post-1970 excerpts.

## Discussion

Presenting excerpts from the post-tonal repertoire, we observed that indications of repetitions in the music in a group of 60 listeners with and without musical training showed significant periods of interindividual synchronization. Ratings of a set of eight questions that probed aspects listeners’ (Q1) familiarity with the piece, (Q2) confidence of repetition responses, and judgments of affordances for (Q3) easy listening, (Q4) coherence, (Q5) similarity of moments, and (Q6) recognition, as well as their (Q7) liking, and (Q8) interest in the piece indicated particularly strong correlations between questions Q2, Q3, and Q4. A PCA on questions Q1 – Q8 and the number of repetition responses as well as the proportion of timepoints with significant inter-participant synchronization of responses suggested that two major factors account for 92% of variance in the data. We interpreted these factors in terms of aesthetic judgment (dominated by Q1, Q7, and Q8) and repetition strength (dominated by the number of repetitions and Q5).

Regression modeling indicated that repetition strength generally differed across excerpt category, with modernist excerpts featuring lower repetition strength compared to post-1970 and tonal excerpts. Aesthetic preference, on the other hand, was significantly lower both for excerpts from modernist and post-1970 pieces compared to excerpts from tonal pieces. The analysis did not reveal any differences in response behavior as a function of musical training with regards to the perception of repetitions, neither as measured by the repetition strength factor, nor for the time series based on the synchronous responses. Note that a comparable independence of musical training and response behavior has been observed in the segmentation literature (Hartmann et al., 2016; Popescu et al., 2021). With regards to Factor 1 (aesthetic preference), however, the regression model indicated a (comparatively weak) interaction effect of excerpt category and musical training, which was based on an association between preference scores for modernist excerpts and the level of musical training of participants, demonstrating a slight rise in preference for post-tonal music by listeners with previous musical training.

### Repetition and Aesthetic Preference as Two Determinants of Post-tonal Music

Previous research in the psycho-aesthetics of music make persistent mention of the co-dependency between complexity and exposure (also sometime called familiarity) and their mediating influence on aesthetic judgments. Based on a hypothesis proposed by Berlyne (1971), the inverted-U model holds that aesthetic preference ratings are likely to be highest when the stimulus is within an optimal intermediate range that lies between the extremities of underdetermined and overly determined complexity. Several studies that examine the effects of familiarity, coherence, and repetition on aesthetic preference judgments have appealed to the inverted-U model to help explain their results after repeated exposures to music with an intervening period of time (Hargreaves, 1984; North and Hargreaves, 1995; Peretz et al., 1998; Tan et al., 2006). Moreover, Margulis (2013) suggests that given the complexity of post-tonal musical structure, and the relative unfamiliarity most participants have with modernist music, the inverted-U model might also help to account for within-composition repetition, as repeated exposure within a limited timeframe may attenuate the perceived complexity of the repeated events. This suggestion conforms to the habituation theory proposed by Huron (2013). A recent review of the music psychology literature asserts the inverted-U model’s efficacy in accounting for a large amount of data (Chmiel and Schubert, 2017).

Taken together, the findings of the present study both complement and challenge earlier work that suggests a link between repetition and aesthetic preference in music listening. In what may be the only previous study devoted exclusively to repetition in post-tonal music, Margulis (2013) showed that aesthetic judgments skew upward when repetitions are inserted into excerpts artificially. It is important to note that participants in study of Margulis (2013) were not asked to identify repetitions, and in many cases were not aware that they had heard multiple versions of the same music containing differing degrees of repetitive materials. Her findings signal the important role repetition plays in listening to music that lacks easily cognized recursion at the structural level and demonstrate that post-tonal music can be made more aesthetically pleasing with the additional perceptual parsing that the repetition of surface events provides.

However, our results may be interpreted to indicate that repetitiveness *per se* is not as strongly correlated to aesthetic preference as the previous literature on both tonal and post-tonal music would seem to indicate. Rather, our data suggests a two-factor model wherein aesthetic preference and repetition strength may be considered to some degree separate determinants of the listening experience. Although none of the excerpts presented within this study both scored highly on the aesthetic preference factor, while at the same time scoring low on the repetition strength factor – a fact that conforms to findings of Margulis (2013) – several observations gleaned from our data converge to demonstrate a degree of independence between repetition and aesthetic preference.

First, there are important discrepancies between the number of repetitions recorded for a given excerpt and the proportion of intersubjective synchronizations for these repeats, demonstrating that not all repetitions are equally perceptually salient (see Figure 2). This finding may also indicate that certain repetitions are perceptually more prominent, and therefore more important to the formal unfolding of a given excerpt than others. Second, synchronous responses do not always correlate with aesthetic qualities (see Figures 2, 3). For instance, “liking” ratings for Tchaikovsky and Grieg were the highest recorded, while the proportion of synchronous responses for the Grieg excerpt are substantially curtailed relative to the Tchaikovsky. Curiously, the confidence self-report for identifying repetitions in the Grieg excerpt is among the highest across excerpts. It is important to note that both of these pieces belong to the tonal category, and therefore the presence of tonal structure cannot account for differences in the results.

Similar discrepancies can be observed for the post-1970 category. For example, the Hurel excerpt displays a large amount and high degree of synchronous responses, but scores relatively low for liking, high for similarity of moments and coherence, and low for ease of following. Conversely, the Dutilleux excerpt displays an extremely low number of repetitions and low participant synchronicity, yet scored moderately well in liking, interest, and ease of following. Although the variety of considered composers and excerpts was naturally limited in this study, these findings may generally be taken to indicate that repetition strength exists somewhat independently of aesthetic preference. As our cross-stylistic results demonstrate, the mere presence of strongly rated repetition does not in itself guarantee aesthetic preference, but in its absence, at least in most cases, aesthetic preference appears to be clearly diminished. Further comparison of the response data together with the quantitative measures for repetition within individual excerpts suggests that additional factors contribute to preference judgments. For example, repetition strength and frequency for the Hurel excerpt would seem to suggest that the music is easy to attend to; repetitions are both prominent and obvious to listeners, at least for the first half of the excerpt. Moreover, ratings for Q5 (similarity) suggest that in general, participants felt the music to be coherent. However, participant ratings for Q3 (easy to follow) and Q4 (coherence) display slightly diminished ratings, and the score for Q7 (liking) reveals the excerpt to not only be the least liked within its category, but also rated on part with the modernist excerpts.

A more pronounced discrepancy is evident when we compare results for the Grisey and Pärt excerpts with those for Grieg and Tchaikovsky, the four excerpts that occupy the upper right quadrant of Figure 4B. First, participant agreement and synchronization are both high and consistent across the Grisey excerpt (Figure 2). From a listener’s perspective, the repeating event can be characterized as low in complexity and evolves only minimally across the excerpt. Repetitions across the excerpt are the most periodic of all the stimuli. For the most part, qualitative response data for Grisey are scored quite high. Questions 2 (repetition confidence), 3 (easy to follow), 4 (coherence), 5 (similarity), and 6 (memorability) all suggest a deeply engaging piece of music. However, relative to the other members of the post-1970 category, interest is flat. And, perhaps more importantly, compared to the two tonal pieces and fellow category member Pärt, Q7 (liking) is rated surprisingly low. Taken together with the lack of development across repetitions, the regularity of the period points to high predictability and rapid habituation as a potential explanation. Excerpts for Pärt and Grieg scored disproportionately high on Q7 (liking) despite receiving far fewer synchronous responses relative to Tchaikovsky, Grisey, and Hurel. In the case of Grieg, the proportion of synchronous is low relative not only to the Tchaikovsky excerpt, but also to Grisey, Hurel, and Pärt. Despite this finding, relatively high scores persist for the Grieg excerpt across all other qualitative measures and culminate in the highest score for liking.

Our data leaves several important questions open to further investigation. The general across-category results would seem to suggest that further underlying parameters play into determinations of aesthetic preference beyond the repetition-liking paradigm hitherto acknowledged by previous literature and supported by the present experiment. Given the structural differences between tonal and post-tonal music, our data suggests that across category differences in responses involves both stylistic considerations and may also involve structural-harmonic differences. However, the relatively consistent categorical differences reported here between what we have termed modernist and post-1970 forms of post-tonal music suggest that further empirical delineation of stylistic, structural, and cognitive elements of post-tonal music are required in order to develop a more complete explanation of aesthetic preference.

With a few important and noteworthy exceptions (McAdams et al., 2004; Poulin-Charronnat et al., 2004), previous research on similarity, repetition, and coherence in Western classical music has limited its stimuli to single instruments. While stimuli for the present study were drawn from the repertoire for ensemble and orchestra, where multiple instrumental parts contribute to complex textures, our experimental design does not take into account the important and intricate sonic differences between simple and complex textures. Moreover, ecological validity of the excerpts was a crucial element in the experimental design that prevents us from testing additional contributing variables such as the differences in harmonic configuration and syntax between tonal and post-tonal music. Although both modernist and post-1970 music are structured using post-tonal configurations, modernist excerpts contain a low number of internal repetitions and score lowest for aesthetic preference. However, post-1970 excerpts, while containing a proportionately larger number of repetitions, score disproportionally lower than tonal excerpts. With the caveat that more research is desirable in order to more comprehensively understand the relationship between repetition and aesthetic preference, and a wider array of compositions inclusive of the plethora of styles that have developed in contemporary music need to be considered, our findings suggest that in addition to repetition, factors including tonality and harmonic configuration, the distribution of repetition, and the specific nature of repeated events, need to be accounted for in research on the perception and cognition of post-tonal music.

## Data Availability Statement

The raw data supporting the conclusions of this article will be made available by the authors, without undue reservation.

## Ethics Statement

The studies involving human participants were reviewed and approved by Kommission für Forschungsfolgenabschätzung und Ethik. The patients/participants provided their written informed consent to participate in this study.

## Author Contributions

MT and KS designed the experiment and co-wrote the manuscript. KS and AK collected and analyzed the data. MT, AK, and KS edited the manuscript. All authors contributed to the article and approved the submitted version.

### Conflict of Interest

The authors declare that the research was conducted in the absence of any commercial or financial relationships that could be construed as a potential conflict of interest.

## Footnotes

^1^www.mathworks.com
