# Supplementary material for: Repetition and Aesthetic Judgment in Post-tonal Music for Large Ensemble and Orchestra
Source: Front Psychol. 2021 Jun 7;12:673706. doi: 10.3389/fpsyg.2021.673706 (PMC8215669; doi:10.3389/fpsyg.2021.673706)
Supplement: Supplementary file 1 [file Data_Sheet_1.PDF]

Supplementary Materials

Repetition and Aesthetic Judgement in Post-tonal Music for Large Ensemble and Orchestra

Moe Touizrar, Anna Lena Knoll, Kai Siedenburg

Self-assessment of musical training

We measured musical training using the corresponding self-report inventory of the Goldsmiths Musical Sophistication Index (Müllensiefen et al., 2014). It includes the following items (with weights of the cumulative index provided in brackets): Years of regular daily practice (1.57), no. of instruments played (0.82), having been complimented on performances [0: never, 1: always] (0.72), no. of hours practiced in period of peak interest (0.71), years of music theory training (1.43), years of instrument training (1.67), considers oneself as a musician [0: fully disagree, 1: fully agree] (0.90).

Statistics from the regression modeling

For computing the PCA, the data from the nine variables (#Rep, and Q1-Q8) were averaged across participants and z-normalized per participant (across excerpts) and per variable. For deriving participant-specific scores along the factors derived from the PCA and the subsequent factor rotation, the data from individual participants was normalized using the same scaling and translation factors per variable as in the z-normalization of the averaged data, before projecting the data on the two rotated factors. The resulting scores of individual participants are plotted in Suppl. Mat. Fig 1.

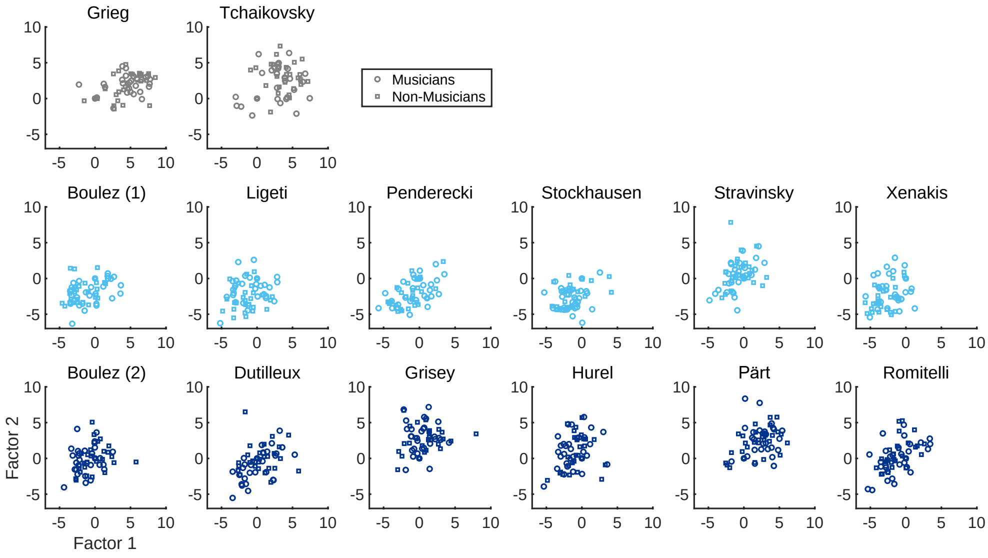

Figure 1 [SM]. Scores of individual participants along Factor 1 (aesthetic preference) and Factor 2 (repetition strength). Circles and squares correspond to participants with and without musical training, respectively.

The LME models contrasted excerpt categories I (tonal) and II (modernist) as well as categories I and III (post-modernist) via dummy coding with category I acting as reference level. The random effects structure of the models included by-participant intercepts and slopes for the category factor as well as by-item intercepts. The dependent variables aesthetic preference (Factor 1) and repetition strength (Factor 2) were z-normalized before entering the model.

Table 3: Statistics from LME for aesthetic preference (Factor 1) with marginal R^2 = 0.36 and conditional R^2 = 0.55. Categories I, II, and III correspond to the group of excerpts from tonal, modernist, and post-modernist pieces, respectively. MSI stands for the Goldsmith Musical Sophistication Index (Müllensiefen et al., 2014) used as a continuous variable in the regression. The notation X : Y corresponds to the interaction factor between variables X and Y.

Aesthetic preference (Factor 1)	β	CI low	CI high	t-value	p-value
Intercept	1.383	0.851	1.915	5.094	< 0.001
Cat. I-II	-1.849	-2.461	-1.237	-5.925	< 0.001
Cat. I-III	-1.377	-1.996	-0.758	-4.358	0.001
MSI	-0.163	-0.348	0.023	-1.719	0.091
Cat. I-II : MSI	0.238	0.031	0.444	2.251	0.028
Cat. I-III: MSI	0.142	-0.086	0.370	1.223	0.226

Table 4: Statistics from LME for repetition strength (Factor 2) with marginal R^2 = 0.31 and conditional R^2 = 0.53. Notation as in Tab. 3.

Repetition strength (Factor 2)	β	CI low	CI high	t-value	p-value
Intercept	0.820	0.177	1.462	2.501	0.029
Cat. I-II	-1.455	-2.201	-0.708	-3.819	0.003
Cat. I-III	-0.458	-1.197	0.281	-1.216	0.249
MSI	-0.024	-0.168	0.119	-0.331	0.741
Cat. I-II : MSI	0.028	-0.157	0.241	0.300	0.766
Cat. I-III : MSI	0.028	-0.124	0.180	0.364	0.716

References

Müllensiefen, D., Gingras, B., Musil, J., and Stewart, L. (2014). The musicality of non-musicians: an index for assessing musical sophistication in the general population. *PLoS ONE,* 9:e89642. doi: 10.1371/journal.pone.0089642
